# Latent TGF-β binding protein 2 and 4 have essential overlapping functions in microfibril development

**DOI:** 10.1038/srep43714

**Published:** 2017-03-02

**Authors:** Yusuke Fujikawa, Hideyuki Yoshida, Tadashi Inoue, Tetsuya Ohbayashi, Kazuo Noda, Harald von Melchner, Toshiji Iwasaka, Ichiro Shiojima, Tomoya O. Akama, Tomoyuki Nakamura

**Affiliations:** 1 Department of Pharmacology, Kansai Medical University, Osaka, 573-1010, Japan; 2 Department of Cardiology, Kansai Medical University, Osaka, 573-1010, Japan; 3 Department of Ophthalmology, Kansai Medical University, Osaka, 753-1010, Japan; 4 Department of Plastic and Reconstructive Surgery, Kansai Medical University, Osaka, 573-1010, Japan; 5 Division of Laboratory Animal Science, Research Center for Bioscience and Technology, Tottori University Graduate School of Medical Sciences, Yonago, Tottori, 683-8503, Japan; 6 Department of Plastic and Reconstructive Surgery, Graduate School of Medicine, Kyoto University, Kyoto, 606-8507, Japan; 7 Department of Molecular Hematology, University of Frankfurt Medical School, Frankfurt am Main, 60590, Germany

## Abstract

Microfibrils are exracellular matrix components necessary for elastic fiber assembly and for suspending lenses. We previously reported that latent TGF-β binding protein 2 (LTBP-2), a microfibril-associated protein, is required for forming stable microfibril bundles in ciliary zonules. However, it was not understood why *Ltbp2* null mice only showed an eye-specific phenotype, whereas LTBP-2 is abundantly expressed in other tissues containing microfibrils in wild type mice. Here, we show that LTBP-4, another microfibril-associated protein, compensates for the loss of LTBP-2 in microfibril formation. *Ltbp2/4S* double knockout (DKO) mice showed increased lethality due to emphysema, which was much more severe than that found in *Ltbp4S* null mice. Elastic fibers in the lungs of *Ltbp2/4S* DKO mice were severely disorganized and fragmented. Cultured mouse embryonic fibroblasts (MEFs) from *Ltbp2/4S* DKO embryos developed reduced microfibril meshwork in serum-free conditions, whereas the microfibril formation was restored by the addition of either recombinant LTBP-2 or -4. Finally, ectopic expression of LTBP-4 in the whole body restored ciliary zonule microfibril bundles in the eyes of *Ltbp2* null mice. These data suggest that LTBP-2 and -4 have critical overlapping functions in forming the robust structure of microfibrils *in vitro* and *in vivo*.

Microfibrils are abundantly distributed extracellular matrix (ECM) components. Some of these serve as a scaffold for tropoelastin deposition to form elastic fibers, whereas others constitute a structural unit without elastin, as in ciliary zonules^1^,^2^. Microfibrils are composed of homopolymers of fibrillin-1 and -2 together with associating proteins such as latent TGF-β binding proteins (LTBPs) and microfibril-associated glycoproteins (MAGPs). Fibrillin-1 and -2 are the defective gene products in Marfan syndrome and congenital contractural arachnodactily, respectively. Microfibrils are organized into various architectures to accommodate the mechanical requirements of different tissues. For example, ciliary zonules in the eye are composed of a thick bundle of microfibrils that conduct tension between the lens and ciliary bodies, whereas in the skin, loosely assembled microfibrils extend from the epidermal basement membrane to the reticular dermis to connect with dermal elastic fibers^3^. In arteries, elastic fibers form a lamellar structure to constitute elastic lamellae. Therefore, microfibrils that provide scaffolds for elastin deposition should also be configured as a lamellar meshwork. The factors that determine the architectures of microfibrils between different tissues remain poorly understood, although microfibril-associating molecules likely play an important role.

LTBPs are multi-domain ECM proteins with molecular masses of 150–220 kDa which share structural homology with fibrillins^4^,^5^,^6^. Among the four LTBPs, LTBP-1 and -3 play major roles in regulating TGF-β bioavailability by tethering TGF-β propeptide (latency-associated peptide or LAP) that binds to mature TGF-β, thus facilitating the secretion, storage in the ECM, and activation of TGF-β. These LTBPs associate with the LAPs of all three TGF-β isoforms; however, LTBP-4 only interacts with TGF-β1-LAP and does so very inefficiently, whereas LTBP-2 does not interact with any TGF-β-LAP complexes^4^,^5^,^6^. Therefore, the primary roles of LTBP-2 and -4 are considered TGF-β-independent.

There have been several reports of human patients with homozygous *LTBP2* null mutations, who suffer from glaucoma with high intraocular pressure (IOP), ectopia lentis, megalocornea and microspherophakia^7^,^8^,^9^,^10^,^11^,^12^. We previously showed that *Ltbp2* null mice exhibit fragmented ciliary zonules leading to lens dislocation^13^. Using cell culture and organ explant culture, we demonstrated that LTBP-2 is required for the formation of stable microfibril bundles in ciliary zonules. However, it is not understood why patients with *LTBP2* mutations or *Ltbp2* null mice only show eye-related phenotypes, whereas LTBP-2 is abundantly expressed in other tissues such as lungs and arteries that are rich in elastic fibers in wild type mice^14^,^15^,^16^.

LTBP-4 plays a major role in elastic fiber development. Homozygous *LTBP4* mutations in humans were reported to cause cutis laxa type 2, a condition characterized by loose skin and emphysema^17^. Mice lacking LTBP-4S, which is the major splicing form expressed in the lungs and intestine, exhibit severely fragmented elastic fibers resulting in emphysematous lungs and stiff arteries^18^. We previously demonstrated that LTBP-4 promotes elastin deposition onto microfibrils through interaction of LTBP-4 with fibulin-5 and fibulin-5 with elastin^19^. However, any role of LTBP-4 in microfibril development is not known.

Here, we show overlapping functions of LTBP-2 and -4 in microfibril development. While *Ltbp4S* null mice show emphysematous lungs and *Ltbp2* null mice have no phenotype in organs other than the eyes, *Ltbp2/4S* DKO mice exhibit a much more severe form of emphysema than that found in *Ltbp4S* null mice, which was associated with increased mortality. The abrogated microfibril formation observed in *Ltbp2/4S* DKO MEFs cultured in serum-free conditions was rescued by the addition of either recombinant LTBP-2 or -4. In addition, ectopic expression of LTBP-4 in *Ltbp2* null mouse eyes restored stable ciliary zonules. This novel function of LTBP-4 in the development of microfibrils, at least in part, explains the absence of an extra-ocular phenotype in *LTBP2*-deficient patients.

## Results

### *Ltbp1* and *3*, but not *4*, mRNA was abundantly expressed in the ciliary bodies of wild type and *Ltbp2* null mice

**Figure 1:**
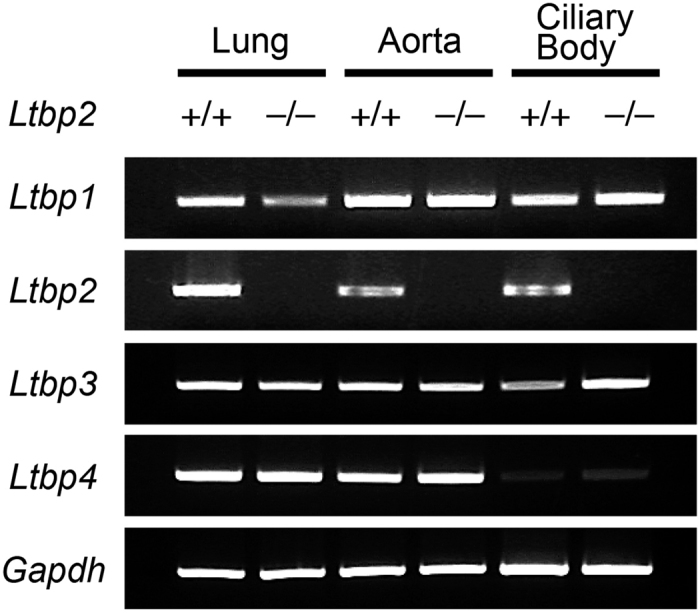
Expression of genes of the LTBP family in mouse tissues. Gene expression of *Ltbp1, 2, 3* and *4* and *Gapdh* in ciliary bodies, aorta and lung tissues from wild type (+/+) and *Ltbp2*^−/−^ (−/−) mice was analyzed using RT-PCR. Expression of *Ltbp4* was hardly detected in ciliary body tissues from either wild type or *Ltbp2*^−/−^ mice.

Assuming that other LTBPs may compensate for the loss of LTBP-2 in stable microfibril formation in tissues other than the eyes in *Ltbp2* null mice, we analyzed the expression of *Ltbp1, 2, 3*, and *4* mRNA in the lungs, aortae, and ciliary bodies of wild type and *Ltbp2* null mice using RT-PCR (Fig. 1). *Ltbp1* and *3* were expressed in ciliary bodies and in other tissues in both wild type and *Ltbp2* null mice, indicating that LTBP-1 and -3 were not able to compensate for the loss of LTBP-2. On the other hand, only a trace amount of *Ltbp4* was expressed in ciliary bodies, while it was abundantly expressed in the lungs and aortae, suggesting that LTBP-4 is a candidate molecule that could substitute for LTBP-2 to enable stable microfibril formation in tissues other than the eyes.

### *Ltbp2/4S* DKO mice had a higher mortality rate than that of *Ltbp2* or *4S* single KO mice

**Figure 2:**
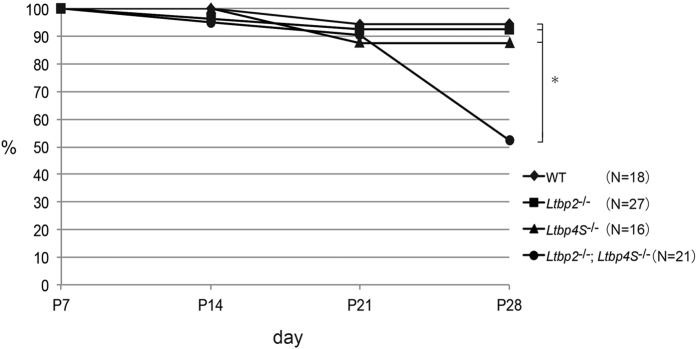
Survival rate of *Ltbp2/4S* mutant mice after birth. Starting from postnatal day 7 (P7), pups of wild type, *Ltbp4S*^−/−^ mice (produced by intercross of *Ltbp4S*^+/−^ mice), *Ltbp2*^−/−^ mice, and *Ltbp2*^−/−^*; Ltbp4S*^−/−^ mice (produced by intercross of *Ltbp2*^−/−^ and *Ltbp4S*^+/−^ mice) were evaluated for survival every week. *P < 0.05, determined by log-rank test.

To assess whether LTBP-4 compensated for the loss of LTBP-2 in *Ltbp2* null mice, we generated *Ltbp2/4S* DKO mice. Since *Ltbp2* null mice do not have any embryonic and reproductive abnormalities, we set up an *Ltbp2*^−/−^; *Ltbp4S*^+/−^ intercross to produce *Ltbp2*^−/−^; *Ltbp4S*^−/−^ (*Ltbp2/4S* DKO) mice. Approximately 50% of *Ltbp2/4S* DKO mice did not survive until the weaning period (4 weeks after birth). We evaluated the survival rate of *Ltbp2/4S* DKO mice along with that of other genotypes in every week after birth (Supplementary Fig. S1a). Mice having a genotype of either *Ltbp2*^−/−^; *Ltbp4S*^+/+^ or *Ltbp2*^−/−^; *Ltbp4S*^+/−^ did not show a major decrease in survival rate at 4 weeks after birth, and most of the *Ltbp2/4S* DKO mice survived by 3 weeks after birth. However, the survival rate of DKO mice suddenly decreased by 50% at 4 weeks after birth (Fig. 2). Dabovic *et al*., reported that *Ltbp4S* null mice show moderate lethality by 4 weeks after birth^20^. However, we analyzed the survival rate of *Ltbp4S* null mice at 4 weeks after birth and did not observe lethality (Fig. 2 and Supplementary Fig. S1b), indicating that juvenile lethality we found in our mutant mice was caused by the cooperative effect of LTBP-2 and LTBP-4 deficiencies. Interestingly, 70% of the *Ltbp2/4S* DKO mice that survived for 4 weeks after birth remained alive for 2 months or more after birth, suggesting a major critical period for survival of *Ltbp2/4S* DKO mice was between 3 weeks and 4 weeks after birth.

### Vascular impairment in *Ltbp2/4S* DKO mice was similar to that found in *Ltbp4S* null mice

To understand the cause of increased lethality in *Ltbp2/4S* DKO mice, we analyzed the physical characteristics and tissue abnormalities in 8-week-old mice of all genotypes, including *Ltbp2/4S* DKO mice. Systolic, diastolic, and mean blood pressure were not different between the genotypes. However, the body weight of *Ltbp2/4S* DKO mice was significantly decreased compared with that of mice of other genotypes (Supplementary Fig. S2).

We reported that *Ltbp4S* null mice show aortic tortuosity at day 5 after birth^19^. We confirmed that neonatal mice of both *Ltbp4S* null and *Ltbp2/4S* DKO genotypes showed tortuous aorta (Supplementary Fig. S3a), although aortic tortuosity became less obvious in adult animals (8-week-old, Supplementary Fig. S3b). Mice with both wild type and *Ltbp2* null genotypes showed no evident aortic abnormalities at the neonatal stage or the adult stage. Although microfibril defects in arteries were expected to result in ascending aortic aneurysm as occurs in Marfan syndrome caused by fibrillin-1 mutations, *Ltbp2/4S* DKO mice did not show apparent aneurysms.

### *Ltbp2/4S* DKO mice show more severe emphysema than that found in *Ltbp4S* null mice

**Figure 3:**
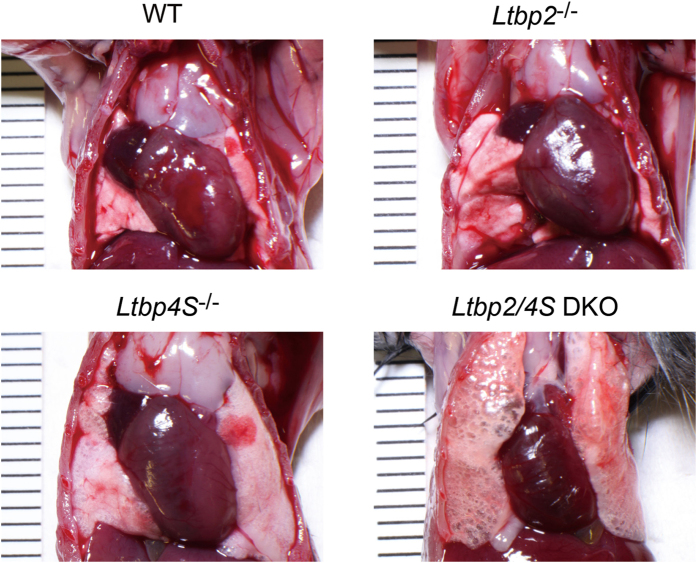
Gross morphology of *Ltbp2/4S* mutant lung in 8-week-old mice. Mild and severe emphysematous phenotypes were observed in *Ltbp4S*^−/−^ and *Ltbp2/4S* DKO mice, respectively.

**Figure 4:**
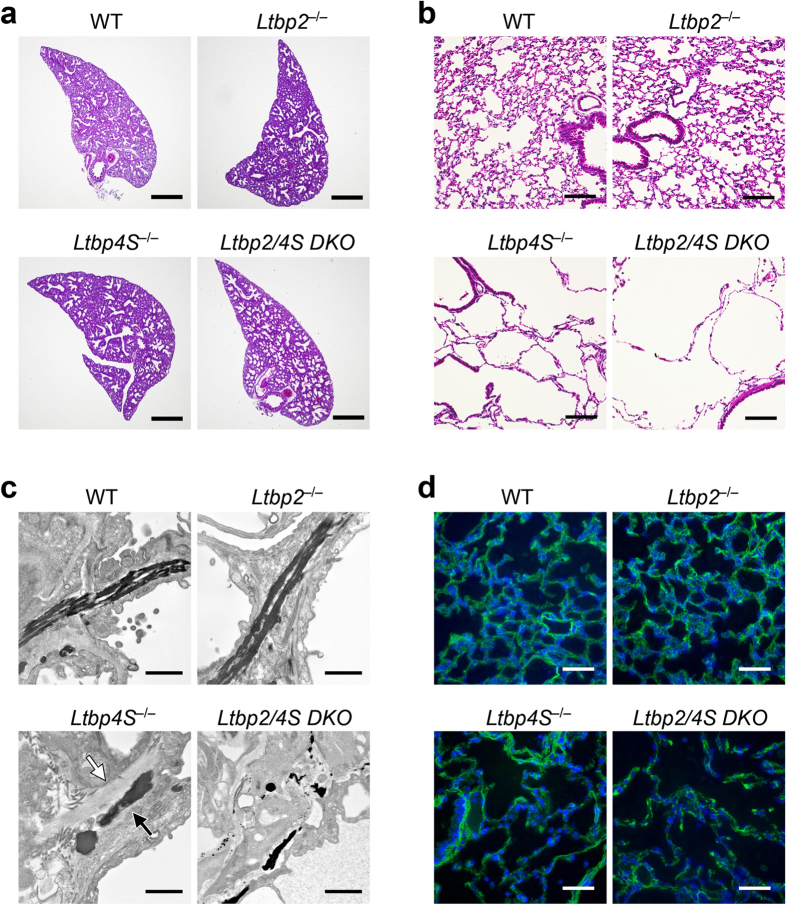
Histological analysis of *Ltbp2/4S* mutant mouse lungs. (**a**) Lung sections of mice at embryonic day 18.5 (E18.5) were stained with hematoxylin and eosin. Bars: 400 μm. (**b**) Lung sections of 8-week-old mice were stained with hematoxylin and eosin. Bars: 100 μm. The terminal air sac enlargement in the lungs of *Ltbp2/4S* DKO mice was more severe than that observed in the lungs of *Ltbp4S*^−/−^ mice. (**c**) Electron micrographs of elastic fibers stained black by tannic acid in the lungs of 8-week-old mice. The elastic fibers of *Ltbp4S*^−/−^ and *Ltbp2/4S* DKO lungs were disorganized. Note bare microfibril bundles (open arrow) and elastin deposits outside microfibrils (closed arrow) in *Ltbp4S*^−/−^ lung tissues. In *Ltbp2/4S* DKO lung tissues, bare microfibril bundles were not observed, and elastic fibers were more severely fragmented. Bars: 1 μm. (**d**) Distribution of microfibrils in the lungs of 8-week-old mice. Cryosections of mouse lungs were stained with a rabbit anti-fibrillin-1 antibody and visualized using an Alexa-488-labeled anti-rabbit IgG antibody. Nuclei were stained with Hoechst 33258. Bars: 50 μm.

*Ltbp4S* null mice were reported to develop pulmonary emphysema^18^,^21^. Macroscopic examination confirmed obvious emphysema in *Ltbp4S* mice, and notably, *Ltbp2/4S* DKO mice showed a more severe form of emphysema than that found in *Ltbp4S* null mice (Fig. 3). Remarkably large terminal air sacs, indicative of disruption of alveolar walls, were observed in the lungs of *Ltbp2/4S* DKO mice (Fig. 3). We then conducted histological analysis of the lungs of wild type and mutant mice at different developmental stages. Lung development in mice proceeds after birth with terminal air sac septation and alveolarization occurring between P0.5 and P21^20^. Before birth, lung morphology was indistinguishable among wild type, *Ltbp2* null, and *Ltbp4S* null mice, whereas the lungs of *Ltbp2/4S* DKO mouse had larger terminal air sacs, indicating a septation defect (Fig. 4a). At 8 weeks after birth, the lungs of wild type and *Ltbp2* null mice showed normal development. However, airspace enlargement was observed in the lungs of adult *Ltbp4S* null mice, and these defects were even more evident in the lung of *Ltbp2/4S* DKO mice (Fig. 4b).

Degradation or impairment of elastic fibers is a major cause of emphysema^22^. Thus, we next analyzed elastic fibers in lung ECM, using electron microscopy. Elastic fibers were visualized by tannic acid staining. Continuous thick elastic fibers were observed along the alveolar walls in wild type and *Ltbp2* null mice (Fig. 4c). These tannic acid-positive elastic fibers were fragmented as dot-like deposits in the lungs of *Ltbp4S* null mice (Fig. 4c). We also observed microfibril bundles devoid of elastin deposition in *Ltbp4S* null tissues, as previously reported^21^, indicating an essential function of LTBP-4 in mediating the proper assembly of elastin on microfibrils^19^. In *Ltbp2/4S* DKO lungs, we only observed smaller pieces of fragmented elastic fibers than in *Ltbp4S* null lung (Fig. 4c). Unlike *Ltbp4S* null tissue, we did not observe microfibril bundles without elastin deposition in *Ltbp2/4S* DKO tissue. Immunofluorescent staining of adult lungs using an anti-fibrillin-1 antibody revealed microfibrils in the wall of alveoli and blood vessels (Fig. 4d). Fibrillin microfibrils in the lungs of *Ltbp2/4S* DKO mice appeared discontinuous compared with those in lung tissues of the other genotypes. These results suggest that the severe emphysematous phenotype observed in *Ltbp2/4S* DKO mice could be attributable to degenerated elastic fibers with inefficient formation of bundled microfibrils, in addition to the terminal air sac septation defect.

### Expression of elastogenic genes was enhanced at 3 weeks after birth but largely reduced at 8 weeks after birth in *Ltbp2/4S* DKO mouse lung tissue

Severe disruption of elastic fibers and alveolar walls in *Ltbp2/4S* DKO mouse lungs suggested impaired elastogenesis and/or an increase in elastolysis in these tissues. Therefore, we analyzed the mRNA expression level of the genes involved in elastic fiber formation and degradation in the lungs at different developmental stages. In neonatal lungs, the expression levels of ECM-related genes were not drastically changed, and the differences from wild type mice were less than 2-fold (Supplementary Fig. S4a). However, at 3 weeks after birth, when *Ltbp2/4S* DKO mice showed partial lethality (Fig. 2), the expression levels of *Eln* and *Fbn2* were increased about 10-fold and 3-fold, respectively, in *Ltbp2/4S* DKO mouse lungs, compared with that of wild type lungs (Supplementary Fig. S4b). The increase in *Fbn1* and *Lox* expression in *Ltbp2/4S* DKO tissues was not statistically significant, but these data indicate compensatory expression of *Eln* but no notable increase in elastolytic metalloproteases. These findings suggested that disruption of elastic fibers was primarily caused by defects in elastic fiber formation in *Ltbp2/4S* DKO lung tissues at this stage, rather than by degradation of elastic fibers. At 8 weeks after birth, which is the period when surviving *Ltbp2/S* DKO mice develop advanced-stage emphysema, *Eln* expression approached the normal level, and expression of other elastogenic genes, including *Fn1, Fbn1, Fbn2, Fbln4, Fbln5*, and *Lox* was dramatically decreased, whereas the expression level of *Mmp12* (macrophage elastase) was increased nearly 3-fold (Supplementary Fig. S4c). These data suggest that elastic fiber-producing mesenchymal cells were impaired and that the MMP-12 produced by macrophages further degraded elastic fibers in *Ltbp2/4S* DKO mouse lung tissues at this stage.

### Either LTBP-2 or -4 is necessary for microfibril formation in serum-free culture of mouse embryonic fibroblasts

**Figure 5:**
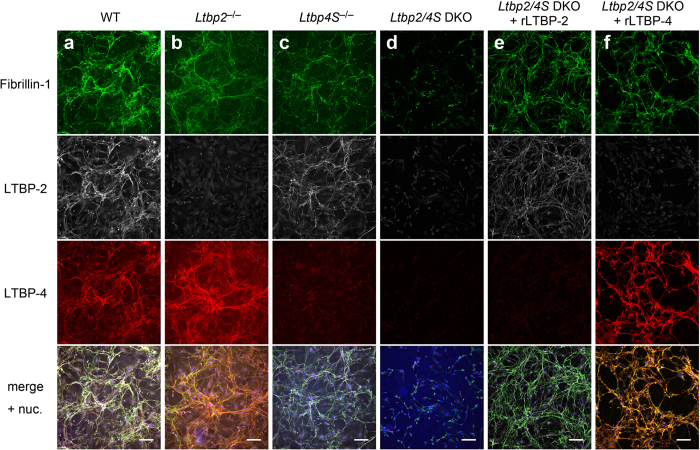
Microfibril formation on cultured MEFs from mutant mice. MEFs of all genotypes were cultured in serum-free media for four days, and the ECM was stained with anti-fibrillin-1, anti-LTBP-2, and anti-LTBP-4 antibodies, followed by fluorophore-labeled secondary antibodies corresponding to each first antibody (green for fibrillin-1, white for LTBP-2, and red for LTBP-4). Nuclei were stained with Hoechst 33258. MEFs lacking either LTBP-2 (**b**) or LTBP-4S (**c**) produced a weak but clear fibrillin-1 meshwork as observed in wild type MEFs (**a**), whereas MEFs lacking both LTBP-2 and LTBP-4S only produced aggregated deposition but no fibrous fibrillin-1–positive meshwork (**d**). Supplementation of recombinant protein of LTBP-2 (**e**) or LTBP-4S (**f**) at a concentration of 5 nM into the cultured medium restored microfibril meshwork formation on *Ltbp2/4S* DKO MEFs. Bars: 150 μm.

To test the hypothesis that LTBP-4 compensates for the loss of LTBP-2 in microfibril formation, we analyzed microfibril formation *in vitro* using cultured mouse embryonic fibroblasts (MEFs) obtained from mutant mouse embryos. MEFs were cultured in serum-free media for 4 days and immunostained with anti-fibrillin-1 (for microfibril formation) as well as anti-LTBP-2 and -4 antibodies. Wild type MEFs produced a fibrillin-1–positive microfibril meshwork on the cells, and *Ltbp2* null MEFs and *Ltbp4S* null MEFs showed slightly reduced signals by anti-fibrillin-1 antibody staining (Fig. 5a–c). Notably, microfibril meshwork formation was significantly abrogated in *Ltbp2/4S* DKO MEFs (Fig. 5d, and Supplementary Fig. S5a), but was rescued by the addition of either 5 nM of recombinant LTBP-2 or -4S (Fig. 5E,F, and Supplementary Fig. S5a). mRNA expression of *Fbn1* and *Fbn2* was even increased in *Ltbp2/4S* DKO MEFs by 2-fold compared to WT MEFs (Supplementary Fig. S5b). These data indicate that either LTBP-2 or LTBP-4S is required for fibrillin-positive microfibril meshwork formation under serum-free conditions. However, microfibril meshwork formation was indistinguishable between the genotypes when cells were cultured in 3% serum-containing medium (Supplementary Fig. S6), suggesting that an unknown element(s) contained in fetal bovine serum promoted microfibril formation without a requirement for endogenous LTBPs. In contrast, elastin deposition onto microfibrils, which was visualized by immunostaining with anti-elastin and anti-fibulin-5 antibodies after cells were cultured in 10% serum-containing media for 14 days, strictly depended on the presence of LTBP-4S protein, with or without LTBP-2 (Supplementary Figs S7 and S8).

### Disruption of ciliary zonules in *Ltbp2* null mice was compensated by ectopic LTBP-4 overexpression

**Figure 6:**
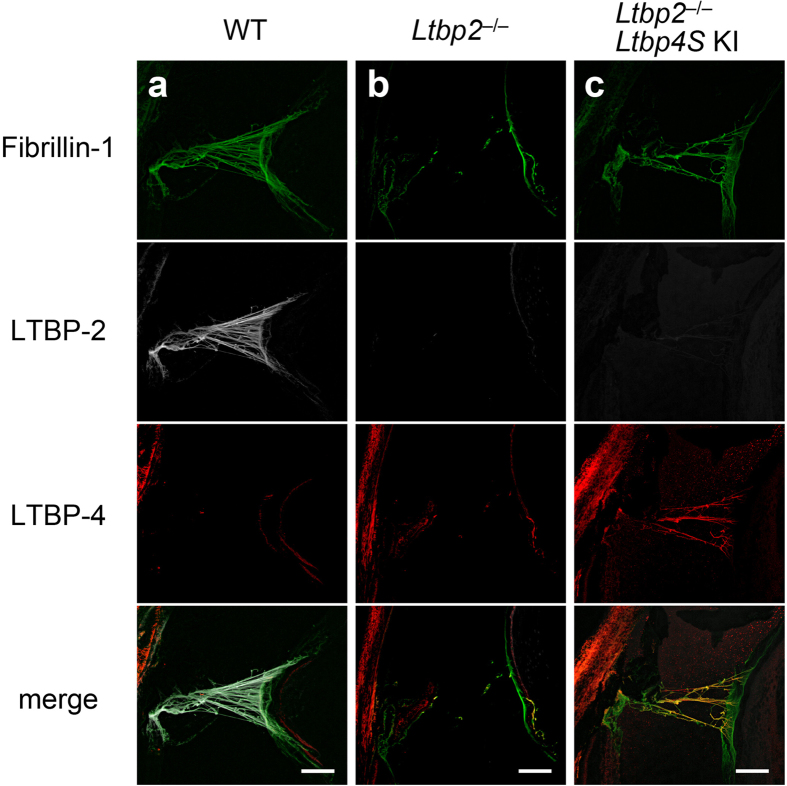
Restoration of ciliary zonule formation in *Ltbp2*^*−/−*^*; Ltbp4S* KI mice. *Ltbp4S* KI mice ubiquitously expressed LTBP-4S in the whole body, including ciliary bodies, whereas LTBP-4 was not expressed in ciliary bodies of wild type mice. By crossing these mice with *Ltbp2*^−/−^ mice, *Ltbp2*^−/−^*; Ltbp4S* KI mice were produced, and ciliary zonule formation of the mouse eyes was visualized using immunofluorescent analysis, as in Fig. 5. LTBP-2 and fibrillin-1 co-localized on ciliary zonules of wild type mouse eyes, but fibrous fibrillin-1 staining was largely absent in the *Ltbp2*^−/−^ eye. Ectopic expression of LTBP-4 in ciliary bodies restored ciliary zonule formation in *Ltbp2*^−/−^*; Ltbp4S* KI mouse eyes. Bars: 100 μm.

Next, we overexpressed LTBP-4 in the eyes of mice with an *Ltbp2* null background, to assess whether LTBP-4 could compensate for the loss of LTBP-2 *in vivo*. Knock-in mice that had a CAG-promoter-driven *Ltbp4S* cDNA at the *Rosa26* locus displayed enhanced expression of *Ltbp4S* in the whole body, and survived to adulthood without any gross malformation (Supplementary Fig. S9). These mice were crossed with *Ltbp2* null mice to obtain *Ltbp2* null; *Ltbp4S* KI mice. Without the *Ltbp4S* knock-in allele, eyes from 4-week-old *Ltbp2* null mice exhibited fragmented ciliary zonules (Fig. 6a,b). However, the zonules were partially restored by the presence of ectopically expressed LTBP-4S in the eyes of *Ltbp2* null; *Ltbp4S* KI mice (Fig. 6c), while LTBP-4S protein co-localized with the fibrillin-1 present in ciliary zonule microfibrils. These data indicated that LTBP-2 and -4 have an overlapping functions in forming the robust architecture of ciliary zonules *in vivo*.

## Discussion

The storage of TGF-β in a latent form in the extracellular space is one of the major functions of LTBPs. However, LTBP-2 and -4 also have TGF-β-independent roles, as LTBP-2 does not bind TGF-β, and only small proportion of LTBP-4 binds TGF-β^4^,^5^,^23^. We and others have reported such TGF-β-independent functions of LTBPs, especially in the development of microfibrils and elastic fibers. LTBP-2 deficiency causes lens luxation due to ciliary zonule fragmentation in humans and mice, suggesting that the function of LTBP-2 is associated with the formation of the robust structure of ciliary zonules consisting of fibrillin-microfibrils^13^. Although LTBP-2 is expressed in tissues other than the eyes that are abundant in microfibrils and elastic fibers, such as the lungs and arteries^15^,^16^, the function of LTBP-2 in these tissues has not been defined, as LTBP-2 deficiency only affected the eyes. On the other hand, we reported that LTBP-4 promotes deposition of elastin onto microfibrils in a TGF-β-independent manner, which is a critical step in elastic fiber assembly, whereas no role of LTBP-4 in microfibril formation has been deteremined^19^. In the current study, we determined that LTBP-2 and LTBP-4 have overlapping functions that enable the production of stable microfibril structures *in vitro* and *in vivo*.

*Ltbp4S* null mice develop pulmonary emphysema and cardiomyopathy^18^. The life span of *Ltbp4S* null mice is variable. Sterner-Kock *et al*. reported that the homozygous mutant mice survived until 6 months of age^18^, whereas Dabovic *et al*. reported a lethality rate of about 30% in homozygous mice by 21 days after birth^20^. In our study, *Ltbp4S* null homozygous mice did not show lethality by at least 4 weeks after birth, consistent with the previous report from 2002^18^. This variation in the survival rate may be due to the different genetic background of the mutant mice.

Whereas *Ltbp2* null mice and *Ltbp4S* null mice did not exhibit increased lethality by 4 weeks after birth, approximately half of the *Ltbp2/4S* DKO mice died at this time point. A more severe form of emphysema than that in *Ltbp4S* null mice was the only critical phenotype we observed that could affect the life span of *Ltbp2/4S* DKO mice. Elastic fibers in the lungs of *Ltbp2/4S* DKO mice were markedly fragmented, which could account for the severe emphysema observed. Because elastic fibers are composed of microfibrils and elastin, and expression of the *Eln* gene was upregulated in the lungs of 3-week-old *Ltbp2/4S* DKO mice, defects in elastic fibers in these tissues were attributed to defects in microfibrils and/or elastin assembly onto microfibrils. Microfibril bundles without elastin deposition were observed in *Ltbp4S* null mice, as previously reported^21^, which is indicative of defective elastin deposition onto microfibrils. The absence of such bare microfibril bundles in *Ltbp2/4S* DKO lungs suggests that the microfibril structure itself was affected in these tissues (Fig. 4c). The fragmented fibrillin-1 staining pattern observed in the lungs of *Ltbp2/4S* DKO mice supports this notion (Fig. 4d). We observed elevated *Mmp12* gene expression in the lungs of surviving 8-week-old *Ltbp2/4S* DKO mice, which could have been caused by chemotactic function of fragmented elastic fibers. At this advanced stage, degradation of elastic fibers by MMP-12 was considered to additively contribute to further impairment in elastic fibers in these tissues.

Another possible explanation is that LTBP-2 and -4 may be protecting microfibrils from enzymatic degradation, rather than forming microfibrils. Further examinations such as time-course of fibrillin-1 immunostaining of lungs and protease resistance assay of microfibrils would elucidate the molecular function of LTBP-2 and -4.

Mutations in the *FBN1* gene cause Marfan syndrome, one of the major symptoms of which is aortic aneurysm^24^. Because fibrillin-1 is the main component of microfibrils, we reasoned that microfibril defects in *Ltbp2/4S* DKO mice could cause aneurysms, which turned out not to be the case. One possibility is that the architecture of microfibrils differs between tissues, and the microfibril architecture in arteries does not require LTBP-2 and -4. Elastic fibers in arteries form a unique structure called elastic lamellae. Therefore, microfibrils that provide scaffolds for elastin deposition could also form a lamellar meshwork that is different from microfibril structures found in the eyes and lungs. Another possible explanation is that aneurysms in Marfan syndrome are caused by a disturbance of signaling maintained by fibrillin-1, and not by defects in microfibril structure per se. It has been reported that antagonizing TGF-β signaling or angiotensin II signaling suppresses the development of aneurysms in *Fbn1* mutant mice or Marfan syndrome patients, even though these drugs does not correct the structural problems in microfibrils caused by mutant fibrillin-1^25^.

Culturing of MEFs required LTBP-2 or -4 for the development of a microfibril meshwork in the absence of fetal bovine serum, although neither of these was necessary for microfibril formation in the presence of serum. These data suggest that circulating LTBP-2, -4, or unknown factors in the serum promoted microfibril formation in the absence of endogenous LTBP-2 and -4. Further study is necessary to identify such circulating factors that facilitate microfibril development.

Ectopically overexpressed LTBP-4 rescued the ciliary zonule phenotype in *Ltbp2* null mice. In *Ltbp2* null mice, ciliary zonule microfibrils developed when the mice were born, but were fragmented after birth, indicating an unstable microfibril structure. We reported an absence of a thick bundle structure of microfibrils in the zonules of these mice, which suggests that LTBP-2 is necessary for the secondary structure of microfibrils in ciliary zonules^13^. The current data demonstrate that LTBP-4 has a similar function.

It has been reported that enhanced TGF-β2 signaling contributes to the lung septation defect in *Ltbp4S* null mice. *Ltbp4S/Tgfb2* DKO mice were shown to have a lessened lung septation defect than that in *Ltbp4S* null mice at E18.5, although elastic fiber impairment was not rescued in the *Ltbp4S/Tgfb2* DKO mice^21^. Although LTBP-2 does not bind TGF-β, and the lung septation defect in *Ltbp4S* null mice at E18.5 was not obvious in our experiments (Fig. 4a), it is possible that the severe emphysematous phenotype in *Ltbp2/4S* DKO mice may also be affected by increased TGF-β signaling. Further study is necessary to examine the role of TGF-β signaling in the pathogenesis of the emphysema present in *Ltbp2/4S* DKO mice.

In summary, we have demonstrated that LTBP-2 and -4 have an overlapping functions in the development of stable microfibril structure *in vivo* in the lungs, eyes, and *in vitro* in MEFs. In ciliary bodies, where LTBP-4 was scarcely expressed, *Ltbp2* deficiency was sufficient to cause disruption of ciliary zonule microfibrils, which was compensated by ectopically overexpressed LTBP-4. Compensation of the microfibril meshwork formation in *Ltbp2/4S* DKO MEFs culture by recombinant LTBP-2 or -4, together with the electron microscopic analyses of mutant lungs also support this notion. Further investigation will elucidate the specific role of LTBP-2 and -4 in the development and organization of microfibril bundle architecture.

## Materials and Methods

### Mice

All mice used in this study were maintained on normal laboratory diet. *Ltbp2*^−/−^ mice were generated with C57BL6/J ES cells and kept in pure C57BL6/J background as previously described^13^. *Ltbp4S*^−/−^ mice^18^ were backcrossed to C57BL6/J more than 10 times before experiments. *Ltbp4S* KI mice, which contain CAG promoter-driven *Ltbp4S* cDNA in the Rosa26 locus (Supplementary Fig. S9), were generated using C57BL6/J ES cells for homologous recombination, and maintained in C57BL6/J background. All procedures were approved by the Committee for Animal Experiments at Kansai Medical University, and conducted according to the Guideline for Animal Experimentation at Kansai Medical University.

### Antibodies and Immunodetection

Primary antibodies used were: chicken anti-LTBP-2 polyclonal (1:100, made by immunizing chickens with recombinant mouse LTBP-2, Figs 5 and 6, Supplementary Figs S6, S7 and S8), goat anti-LTBP-4 polyclonal (1:100; R&D Systems, Figs 5 and 6, Supplementary Figs S6, S7 and S8), rabbit anti-mouse fibrillin-1 polyclonal (1:100, previously described^13^, Figs 4–6, Supplementary Fig. S6), rabbit anti-elastin polyclonal PR385 (1:100; Elastin Products Company), and mouse anti-fibulin-5 monoclonal 10A (1:100, previously described^26^, Supplementary Fig. S8). Secondary antibodies conjugated with Alexa 488, 546 and Dylight 650 were purchased from Thermo Fisher Scientific (Alexa 488 and 546) and Leinco Technologies (Dylight 650). Frozen sections were fixed with acetone at −20 °C for 5 min, blocked with PBS containing 1% bovine serum albumin and 0.05% Triton X-100 before immunofluorescent staining. Immunofluorescence images were obtained using all-in-one fluorescence microscope BZ-9000 (Keyence).

### Cell Culture

MEFs were maintained in DMEM (Thermo Fisher Scientific) supplemented with 2 mM glutamine, 100 units/100 μg·mL^−1^ penicillin/streptomycin, and 10% FBS at 37 °C in 5% CO_2_. For microfibril analyses, MEFs were cultured on coverslips in DMEM/F12 (Thermo Fisher Scientific) supplemented with 2 mM glutamine, 100 units/100 μg·mL^−1^ penicillin/streptomycin, 1x ITS-X (Thermo Fisher Scientific) with or without 3% FBS at 37 °C in 5% CO_2_ for 4 days, followed by fixation with cold methanol and immunostaining. For elastic fiber analyses, 10% FBS instead of ITS-X was used and culture period was extended to 14 days.

### Protein Purification

Purification of recombinant LTBP-2 and -4S with a FLAG and a 6x His tag was described previously^19^,^26^. Briefly, these proteins were purified using TALON affinity resin (Takara) from serum-free conditioned medium of 293T cells stably transfected with pEF6/FLAG-LTBP-2 or -4S.

### Electron Microscopy

Lung samples were fixed with 2% paraformaldehyde, 2% glutaraldehyde in 0.1 M cacodylate buffer (pH 7.4) at 4 °C overnight. The samples were additionally stained with 1% tannic acid in 0.1 M cacodylate buffer 1 hour, followed by wash and post-fixation with 2% osmium tetroxide in 0.1 M cacodylate buffer. The samples were embedded in resin Quetol-812 (Nisshin EM). Ultra-thin sections were stained with 2% uranyl acetate and lead stain solution (Sigma-Aldrich), and were observed with transmission electron microscope JEM-1400plus (JEOL Ltd.).

### RT-PCR and qPCR

Total RNA from tissues and MEFs was extracted using TRIzol reagent (Thermo Fisher Scientific) and RNeasy kit (Qiagen), respectively, and transcribed to cDNA with random hexamers using SuperScript III First-Strand Synthesis System (Thermo Fisher Scientific). RT-PCR was performed using the primers indicated in Supplementary Table S1. For qPCR, the reaction was performed with QuantiFast SYBR Green PCR Kit (Qiagen), and the products were analyzed with Rotor-Gene Q (Qiagen). The primer sequences used for qPCR are shown in Supplementary Table S2.

### Quantification of microfibrils produced by MEFs

MEFs cultured on 96-well optical bottom plate (Thermo Fisher Scientific) were fixed with cold methanol and reacted with anti-fibrillin-1 antibody, followed by staining with Alexa 488-conjugated secondary antibody and Hoechst nuclear dye. The amount of immunostained microfibrils and cell numbers were measured with Cellomics ArrayScan VTI system (Thermo Fisher Scientific).

### Statistics

Statistical analysis of survival rate was performed by log-rank test. Statistical analyses of qPCR, quantification of microfibrils, and physical characteristics were performed by one-way ANOVA with Dunnett’s post hoc test. P < 0.05 compared to WT was considered significant.

## Additional Information

**How to cite this article:** Fujikawa, Y. *et al*. Latent TGF-β binding protein 2 and 4 have essential overlapping functions in microfibril development. *Sci. Rep.* **7**, 43714; doi: 10.1038/srep43714 (2017).

**Publisher's note:** Springer Nature remains neutral with regard to jurisdictional claims in published maps and institutional affiliations.

## Supplementary Material

Supplementary Information

## References

[b1] Ramirez F., Sakai L. Y., Dietz H. C. & Rifkin D. B. Fibrillin microfibrils: multipurpose extracellular networks in organismal physiology. Physiol Genomics 19, 151–154 (2004). 15466717 10.1152/physiolgenomics.00092.2004

[b2] Kielty C. M., Sherratt M. J., Marson A. & Baldock C. Fibrillin microfibrils. Adv Protein Chem 70, 405–436 (2005). 15837522 10.1016/S0065-3233(05)70012-7

[b3] Kielty C. M., Sherratt M. J. & Shuttleworth C. A. Elastic fibres. J Cell Sci 115, 2817–2828 (2002). 12082143 10.1242/jcs.115.14.2817

[b4] Koli K., Saharinen J., Hyytiäinen M., Penttinen C. & Keski-Oja J. Latency, activation, and binding proteins of TGF-beta. Microsc Res Tech 52, 354–362 (2001). 11170294 10.1002/1097-0029(20010215)52:4<354::AID-JEMT1020>3.0.CO;2-G

[b5] Rifkin D. B. Latent transforming growth factor-beta (TGF-beta) binding proteins: orchestrators of TGF-beta availability. J Biol Chem 280, 7409–7412 (2005). 15611103 10.1074/jbc.R400029200

[b6] Robertson I. B. . Latent TGF-β-binding proteins. Matrix Biol 47, 44–53 (2015). 25960419 10.1016/j.matbio.2015.05.005 PMC4844006

[b7] Ali M. . Null mutations in LTBP2 cause primary congenital glaucoma. Am J Hum Genet 84, 664–671 (2009). 19361779 10.1016/j.ajhg.2009.03.017 PMC2680998

[b8] Narooie-Nejad M. . Loss of function mutations in the gene encoding latent transforming growth factor beta binding protein 2, LTBP2, cause primary congenital glaucoma. Hum Mol Genet 18, 3969–3977 (2009). 19656777 10.1093/hmg/ddp338

[b9] Azmanov D. N. . LTBP2 and CYP1B1 mutations and associated ocular phenotypes in the Roma/Gypsy founder population. Eur J Hum Genet 19, 326–333 (2011). 21081970 10.1038/ejhg.2010.181 PMC3062003

[b10] Désir J. . LTBP2 null mutations in an autosomal recessive ocular syndrome with megalocornea, spherophakia, and secondary glaucoma. Eur J Hum Genet 18, 761–767 (2010). 20179738 10.1038/ejhg.2010.11 PMC2987369

[b11] Kumar A. . A homozygous mutation in LTBP2 causes isolated microspherophakia. Hum Genet 128, 365–371 (2010). 20617341 10.1007/s00439-010-0858-8

[b12] Khan A. O., Aldahmesh M. A. & Alkuraya F. S. Congenital megalocornea with zonular weakness and childhood lens-related secondary glaucoma - a distinct phenotype caused by recessive LTBP2 mutations. Mol Vis 17, 2570–2579 (2011). 22025892 PMC3198484

[b13] Inoue T. . Latent TGF-β binding protein-2 is essential for the development of ciliary zonule microfibrils. Hum Mol Genet 23, 5672–5682 (2014). 24908666 10.1093/hmg/ddu283 PMC4189902

[b14] Gibson M. A. . Bovine latent transforming growth factor beta 1-binding protein 2: molecular cloning, identification of tissue isoforms, and immunolocalization to elastin-associated microfibrils. Mol Cell Biol 15, 6932–6942 (1995). 8524260 10.1128/mcb.15.12.6932 PMC230948

[b15] Shipley J. M. . Developmental expression of latent transforming growth factor beta binding protein 2 and its requirement early in mouse development. Mol Cell Biol 20, 4879–4887 (2000). 10848613 10.1128/mcb.20.13.4879-4887.2000 PMC85939

[b16] Hirai M. . Latent TGF-beta-binding protein 2 binds to DANCE/fibulin-5 and regulates elastic fiber assembly. EMBO J 26, 3283–3295 (2007). 17581631 10.1038/sj.emboj.7601768 PMC1933399

[b17] Urban Z. . Mutations in LTBP4 cause a syndrome of impaired pulmonary, gastrointestinal, genitourinary, musculoskeletal, and dermal development. Am J Hum Genet 85, 593–605 (2009). 19836010 10.1016/j.ajhg.2009.09.013 PMC2775835

[b18] Sterner-Kock A. . Disruption of the gene encoding the latent transforming growth factor-beta binding protein 4 (LTBP-4) causes abnormal lung development, cardiomyopathy, and colorectal cancer. Genes Dev 16, 2264–2273 (2002). 12208849 10.1101/gad.229102 PMC186672

[b19] Noda K. . Latent TGF-β binding protein 4 promotes elastic fiber assembly by interacting with fibulin-5. Proc Natl Acad Sci USA 110, 2852–2857 (2013). 23382201 10.1073/pnas.1215779110 PMC3581912

[b20] Dabovic B. . Control of lung development by latent TGF-β binding proteins. J Cell Physiol 226, 1499–1509 (2011). 20945348 10.1002/jcp.22479 PMC3060286

[b21] Dabovic B. . Dual functions for LTBP in lung development: LTBP-4 independently modulates elastogenesis and TGF-beta activity. J Cell Physiol 219, 14–22 (2009). 19016471 10.1002/jcp.21643 PMC2719250

[b22] Shifren A. & Mecham R. P. The stumbling block in lung repair of emphysema: elastic fiber assembly. Proc Am Thorac Soc 3, 428–433 (2006). 16799087 10.1513/pats.200601-009AW PMC2658707

[b23] Todorovic V. & Rifkin D. B. LTBPs, more than just an escort service. J Cell Biochem 113, 410–418 (2012). 22223425 10.1002/jcb.23385 PMC3254144

[b24] Ramirez F. & Dietz H. C. Marfan syndrome: from molecular pathogenesis to clinical treatment. Curr Opin Genet Dev 17, 252–258 (2007). 17467262 10.1016/j.gde.2007.04.006

[b25] Matt P. . Recent advances in understanding Marfan syndrome: should we now treat surgical patients with losartan? J Thorac Cardiovasc Surg 135, 389–394 (2008). 18242274 10.1016/j.jtcvs.2007.08.047

[b26] Hirai M. . Fibulin-5/DANCE has an elastogenic organizer activity that is abrogated by proteolytic cleavage *in vivo*. J Cell Biol 176, 1061–1071 (2007). 17371835 10.1083/jcb.200611026 PMC2064089

