# Supplementary material for: Latent TGF-β binding protein 2 and 4 have essential overlapping functions in microfibril development
Source: Sci Rep. 2017 Mar 2;7:43714. doi: 10.1038/srep43714 (PMC5333096; doi:10.1038/srep43714)
Supplement: Supplementary Information [file srep43714-s1.pdf]

Supplementary Information

Latent TGF-β binding protein 2 and 4 have essential overlapping functions in microfibril development

Yusuke Fujikawa[1,2], Hideyuki Yoshida[1,3], Tadashi Inoue[1,4], Tetsuya Ohbayashi[5], Kazuo Noda[6], Harald von Melchner[7], Toshiji Iwasaka[2], Ichiro Shiojima[2], Tomoya O. Akama[1], Tomoyuki Nakamura[1,*]

Department of [1]Pharmacology, [2]Cardiology, [3]Ophthalmology, and [4]Plastic and Reconstructive Surgery, Kansai Medical University, Osaka, 573-1010, Japan

[5]Division of Laboratory Animal Science, Research Center for Bioscience and Technology, Tottori University Graduate School of Medical Sciences, Yonago, Tottori, 683-8503, Japan

[6]Department of Plastic and Reconstructive Surgery, Graduate School of Medicine, Kyoto University, Kyoto, 606-8507, Japan

[7]Laboratory for Molecular Hematology, University of Frankfurt Medical School, Frankfurt am Main, 60590, Germany

[*]Address correspondence to:
Tomoyuki Nakamura, MD, PhD
nakamtom@hirakata.kmu.ac.jp
2-5-1 Shinmachi, Hirakata, Osaka 573-1010, Japan

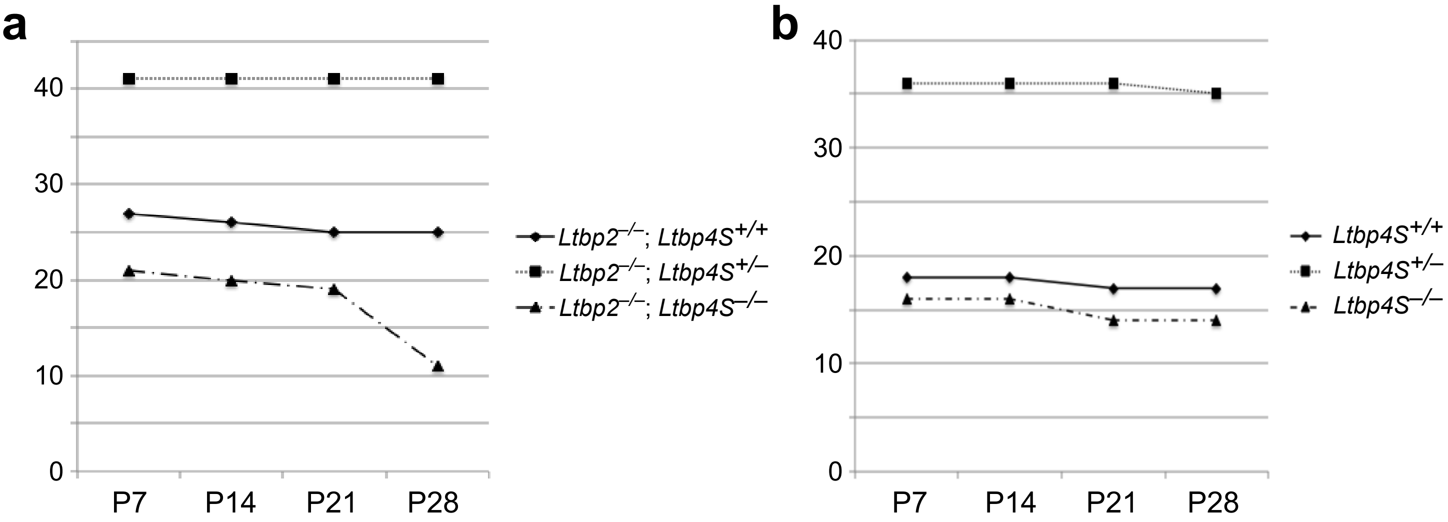

Supplementary Figure S1. Survival of *Ltbp4S*$^{+/+}$, *Ltbp4S*$^{+/-}$ and *Ltbp4S*$^{-/-}$ mice in *Ltbp2*$^{-/-}$ (a) or wild type (b) background. Newborn mice were produced from intercross of either *Ltbp2*$^{-/-}$;*Ltbp4S*$^{+/-}$ (a) or *Ltbp4S*$^{+/-}$ (b). Number of live mice at every week after birth was plotted on the graphs by postnatal day 28 (P28).

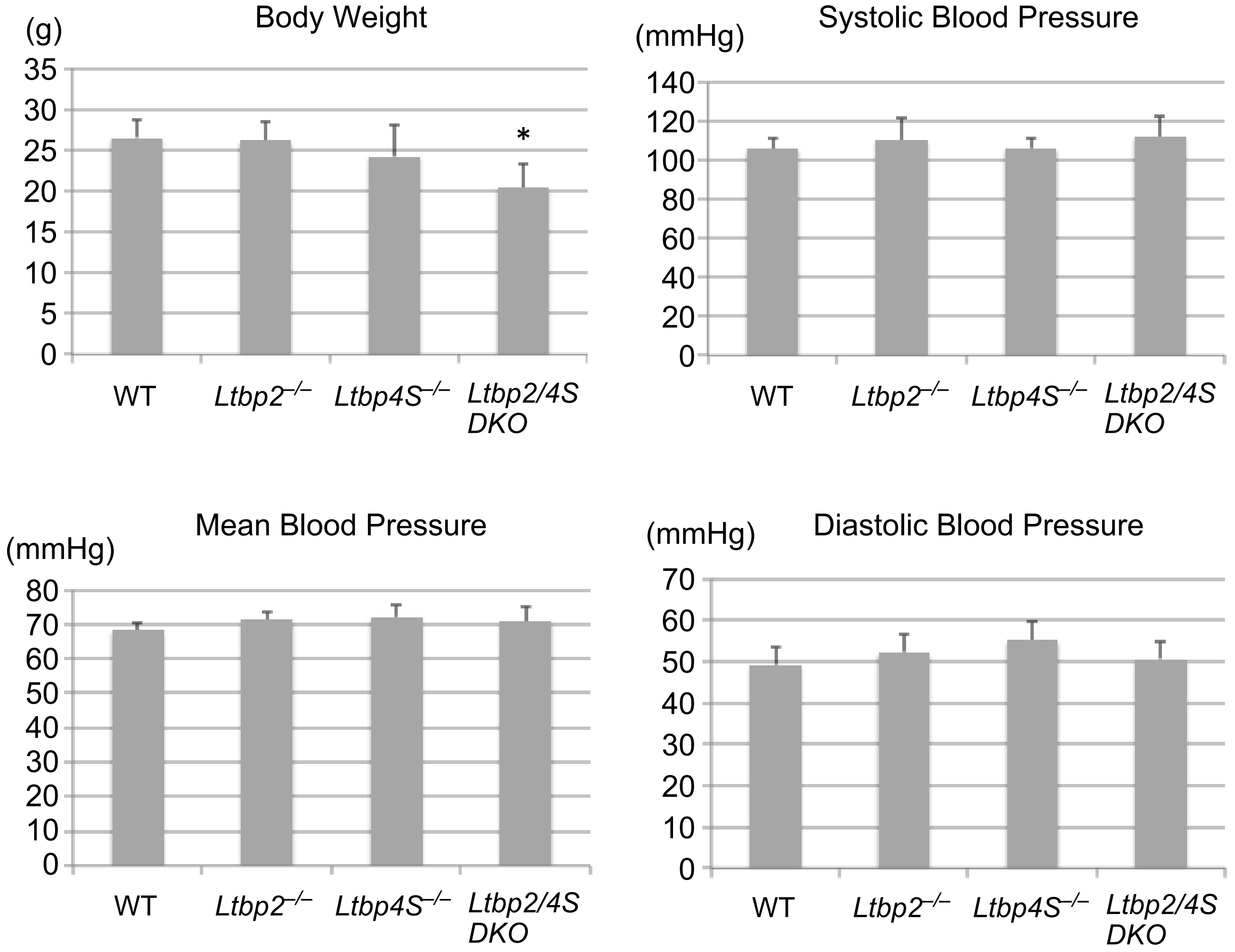

Supplementary Figure S2. Physical characteristics of wild type, *Ltbp2*$^{-/-}$, *Ltbp4S*$^{-/-}$ and *Ltbp2/4S* DKO mice at 8 weeks old. Ten mice were analyzed in each genotype. Data are expressed as mean ± SEM (parametric results). Body weight (top left), systolic blood pressure (top right), mean blood pressures (bottom left) and diastolic blood pressure (bottom right) were measured. *$P < 0.05$, compared to WT.

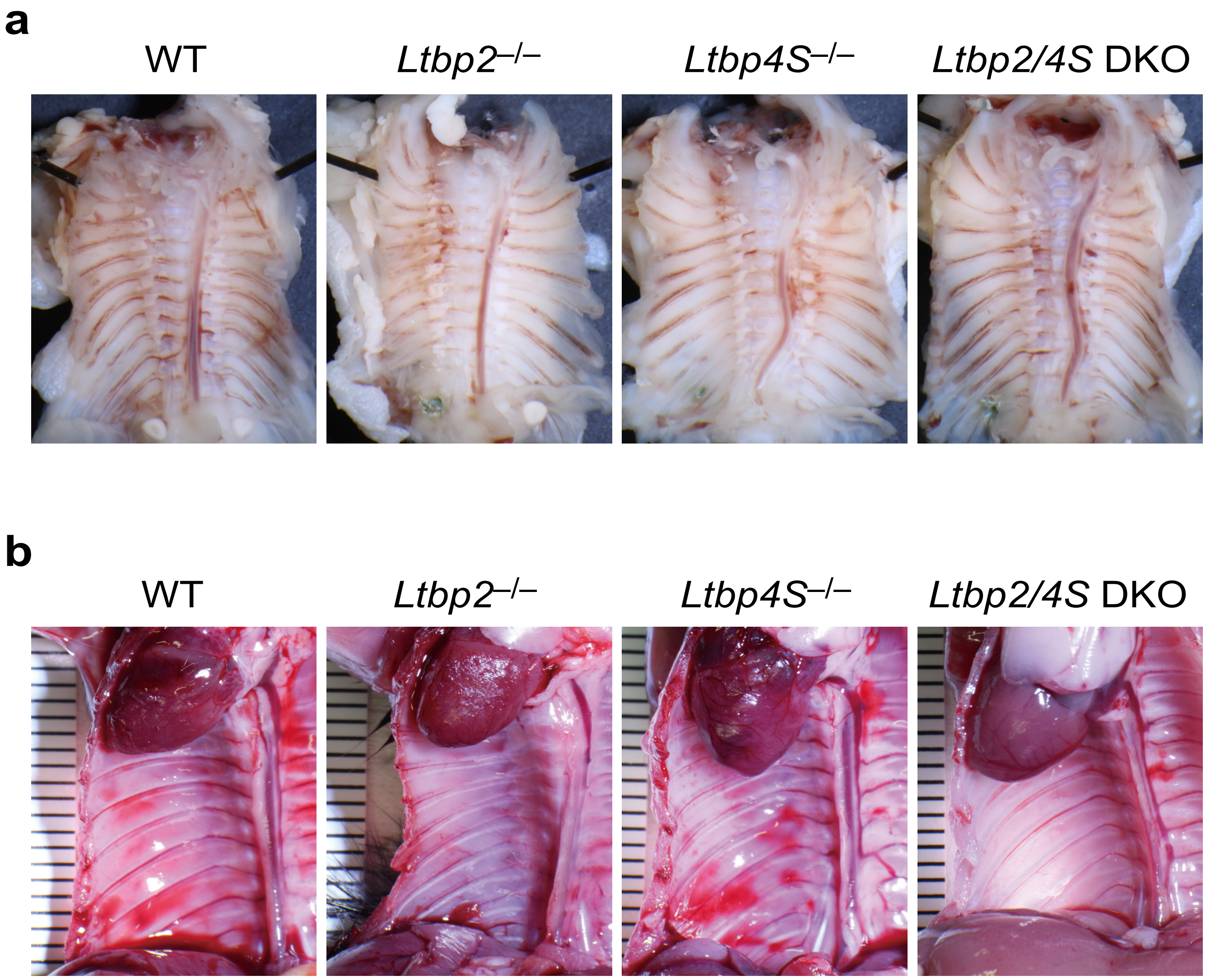

Supplementary Figure S3. Gross morphology of *Ltbp2/4S* mutant aorta in P0.5 (a) and 8-week-old (b) mice. Aortae of *Ltbp4S*$^{-/-}$ and *Ltbp2/4S* DKO mice were tortuous at newborn stage, but the tortuosity was not obvious at adult stage.

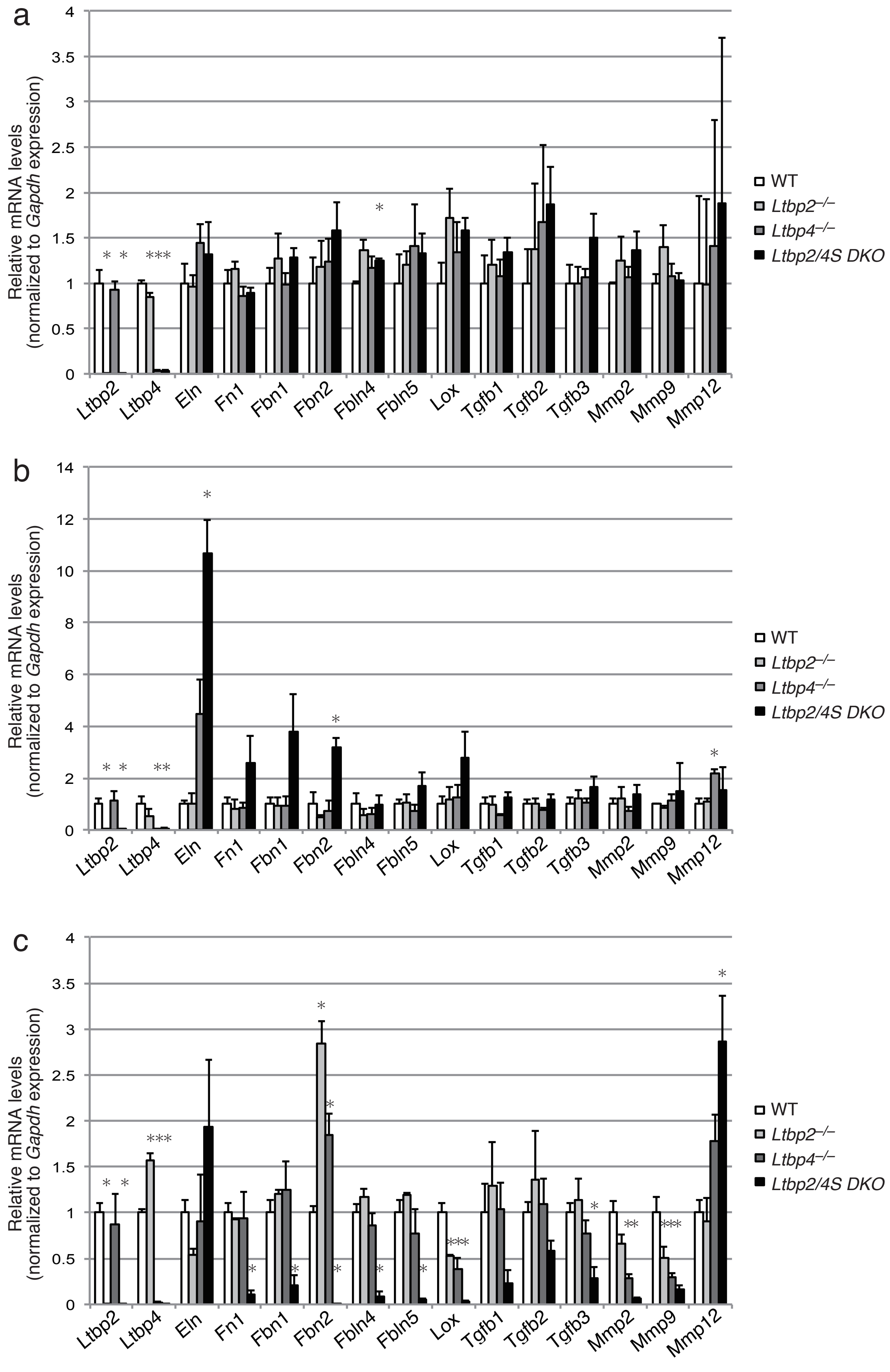

Supplementary Figure S4. Expression of genes involved in elastogenesis in mouse lung. Relative gene expression in mouse lungs of all genotypes was assessed by quantitative RT-PCR (three lungs were used per genotype). Expression level of each gene was standardized to expression levels of a housekeeping gene, *Gapdh*. Data are expressed as mean ± SEM. n=4, $*P<0.05$ compared to WT. **a.** Expression of elastogenesis-related genes in neonatal (P0.5) mouse lungs. **b.** Expression of elastogenesis-related genes in young (P21) mouse lungs. Note that mRNA level of Tropoelastin was increased more than 10-fold in *Ltbp2/4S* DKO lung compared to that in wild type. **c.** Expression of elastogenesis-related genes in adult (8-week-old) mouse lungs. Expression level of MMP-12 gene was increased remarkably in *Ltbp2/4S* DKO mice lung compared to the other lungs.

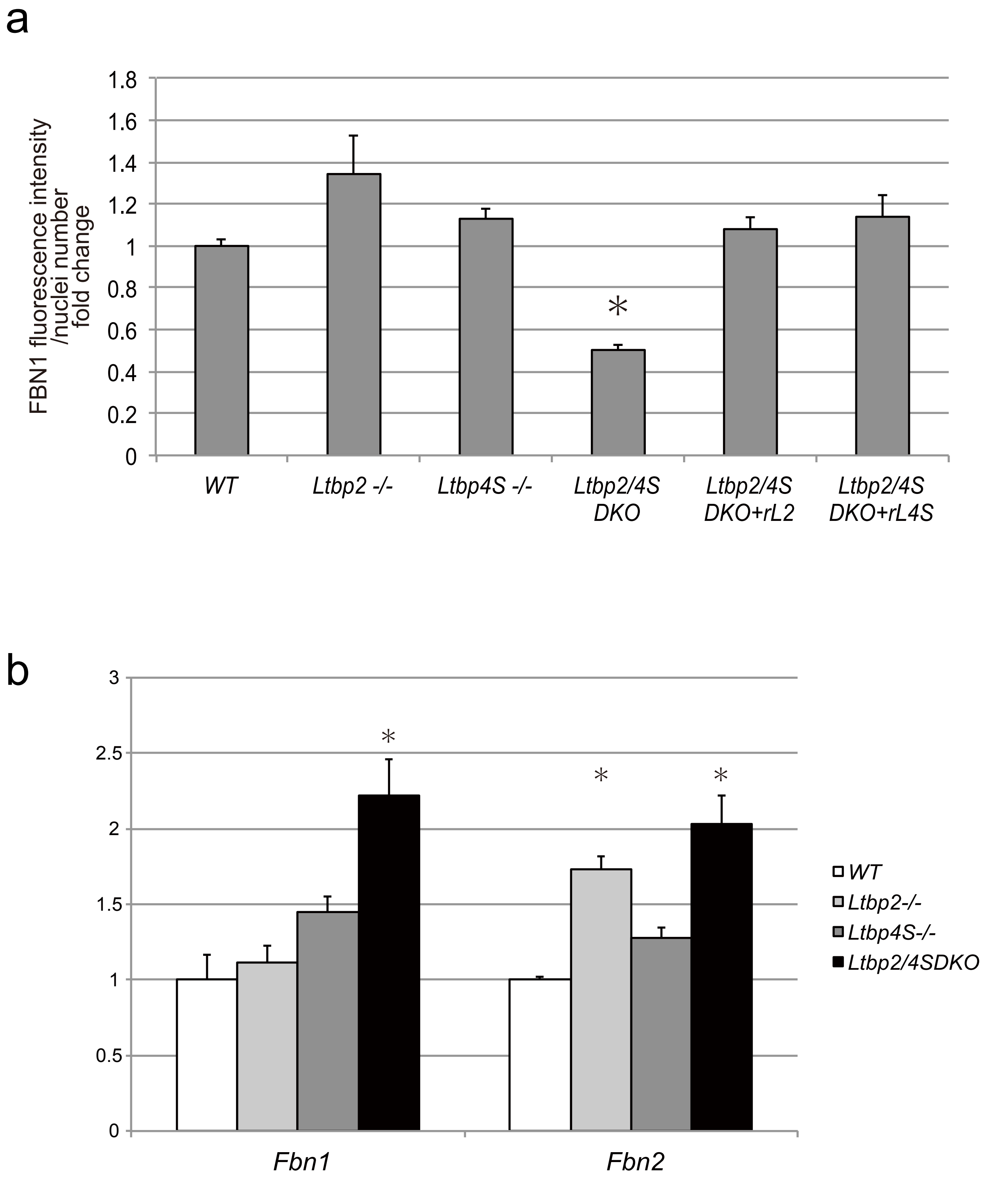

Supplementary Figure S5. Quantitative analysis of fibrillin-microfibrils (a) and fibrillins mRNA (b) in serum-free MEF cultures. **a.** Fluorescence intensity of immunostained Fibrillin-1 produced by MEFs and nuclei number were measured using Cellomics ArrayScan VTI system (Thermo Fisher Scientific). Fluorescence intensities divided by nuclei numbers were compared. n=5, *p<0.05 compared to WT. **b.** mRNA expression of *Fbn1* and *Fbn2* was measured by qPCR. *Fbn1* and *Fbn2* expression was significantly increased in *Ltbp2/4S* DKO MEFs compared to WT MEFs. n=3, *p<0.05 compared to WT.

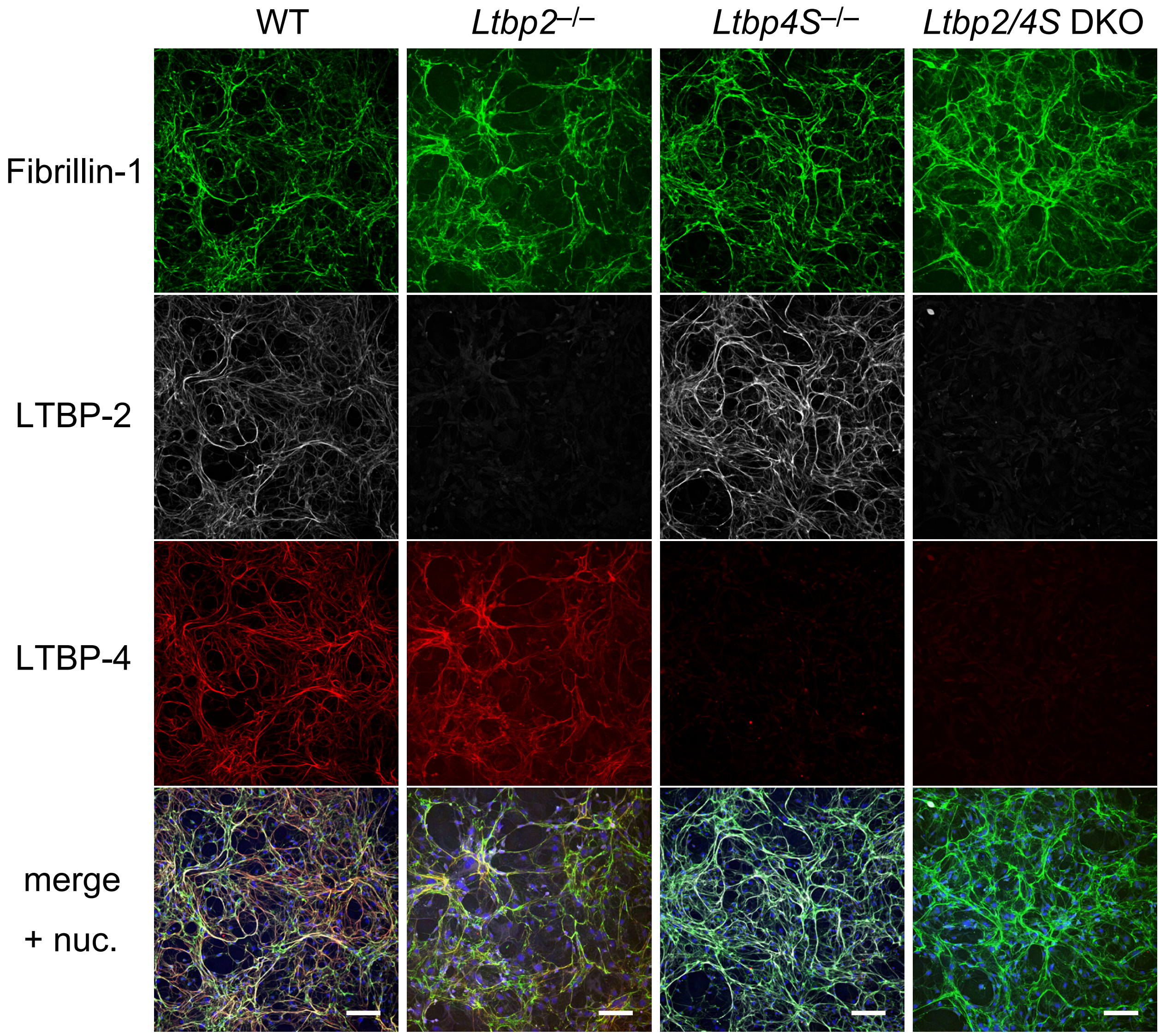

Supplementary Figure S6. Microfibril formation on mutant MEFs cultured in serum-supplemented medium. MEFs of all genotypes were cultured in 3% serum-contained media for four days, and then formation of extracellular matrix on the cells was evaluated with immunohistochemical analysis as in Figure 5. Fibrillin-1 fiber meshwork was detected normally in all MEFs. Bars: 150 μm.

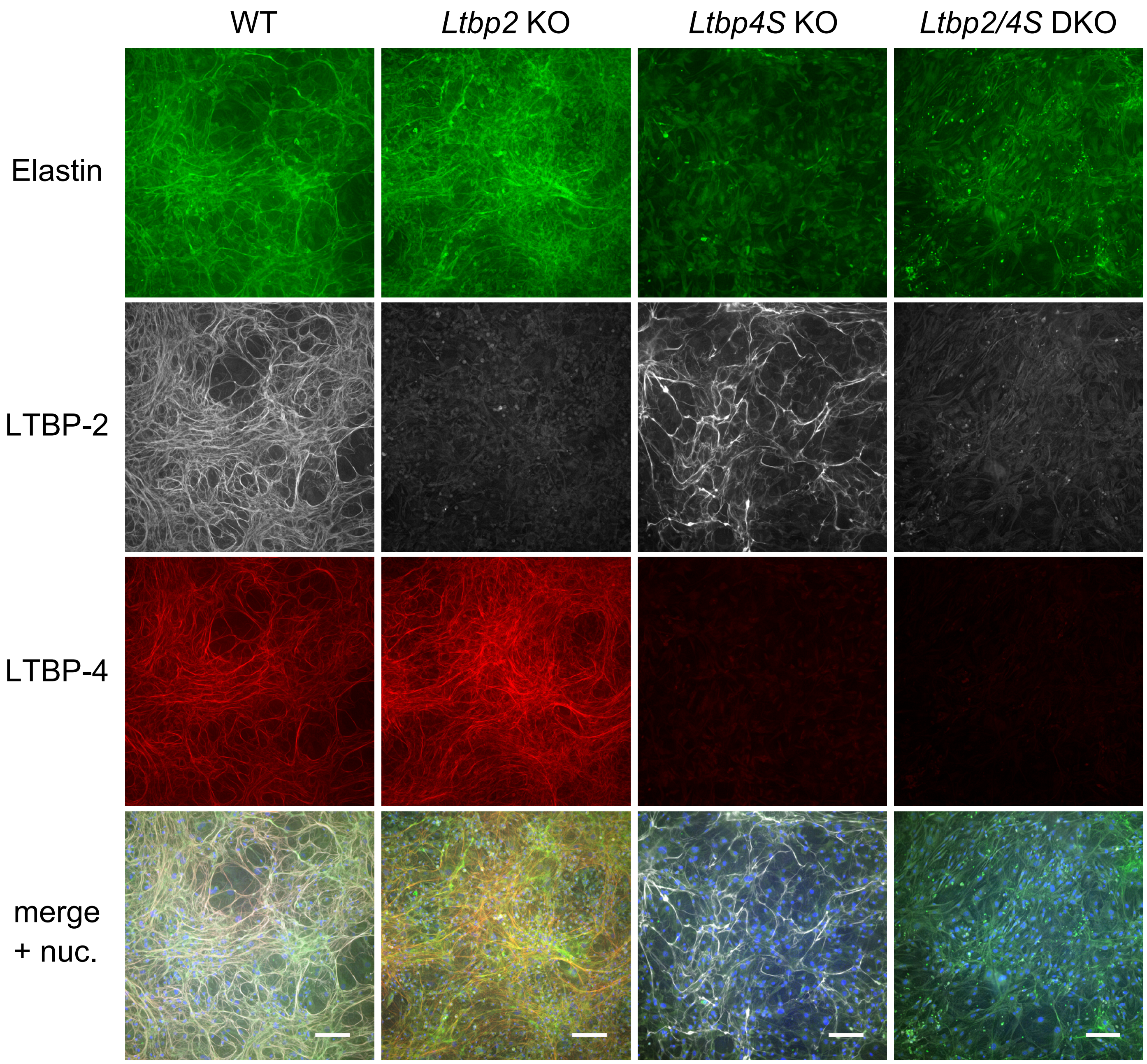

Supplementary Figure S7. Fibrous deposition of elastin on mutant MEFs. MEFs were cultured in 10% serum-contained media for 14 days, and were stained with anti-elastin (Elastin, first row), anti-LTBP-2 (LTBP-2, second row) and anti-LTBP-4 (LTBP-4, third row) antibodies, followed by fluorophore-labeled secondary antibodies corresponding to each first antibody (green for elastin, white for LTBP-2, red for LTBP-4). Regardless of presence of LTBP-2, MEFs lacking LTBP-4 did not produce assembled elastic fiber meshwork. Bars: 150 μm.

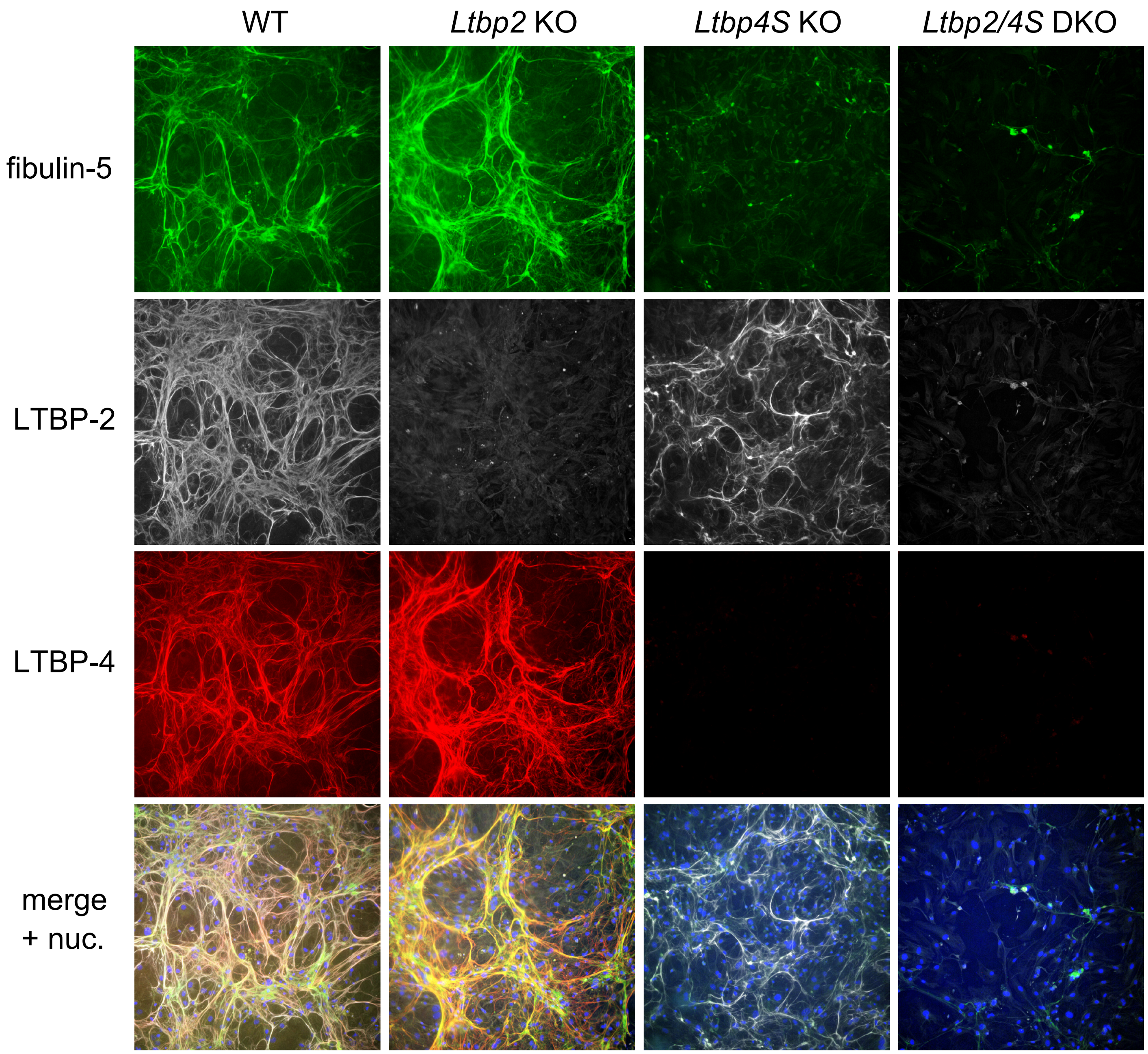

Supplementary Figure S8. Fibrous deposition of fibulin-5 on mutant MEFs. MEFs were cultured in 10% serum-contained media for 14 days, and were stained with anti-fibulin-5 (fibulin-5, first row), anti-LTBP-2 (LTBP-2, second row) and anti-LTBP-4 (LTBP-4, third row) antibodies, followed by fluorophore-labeled secondary antibodies corresponding to each first antibody (green for fibulin-5, white for LTBP-2, red for LTBP-4). Similar to elastin deposition, fibulin-5 did not deposit on microfibrils without LTBP-4. Bars: 150 μm.

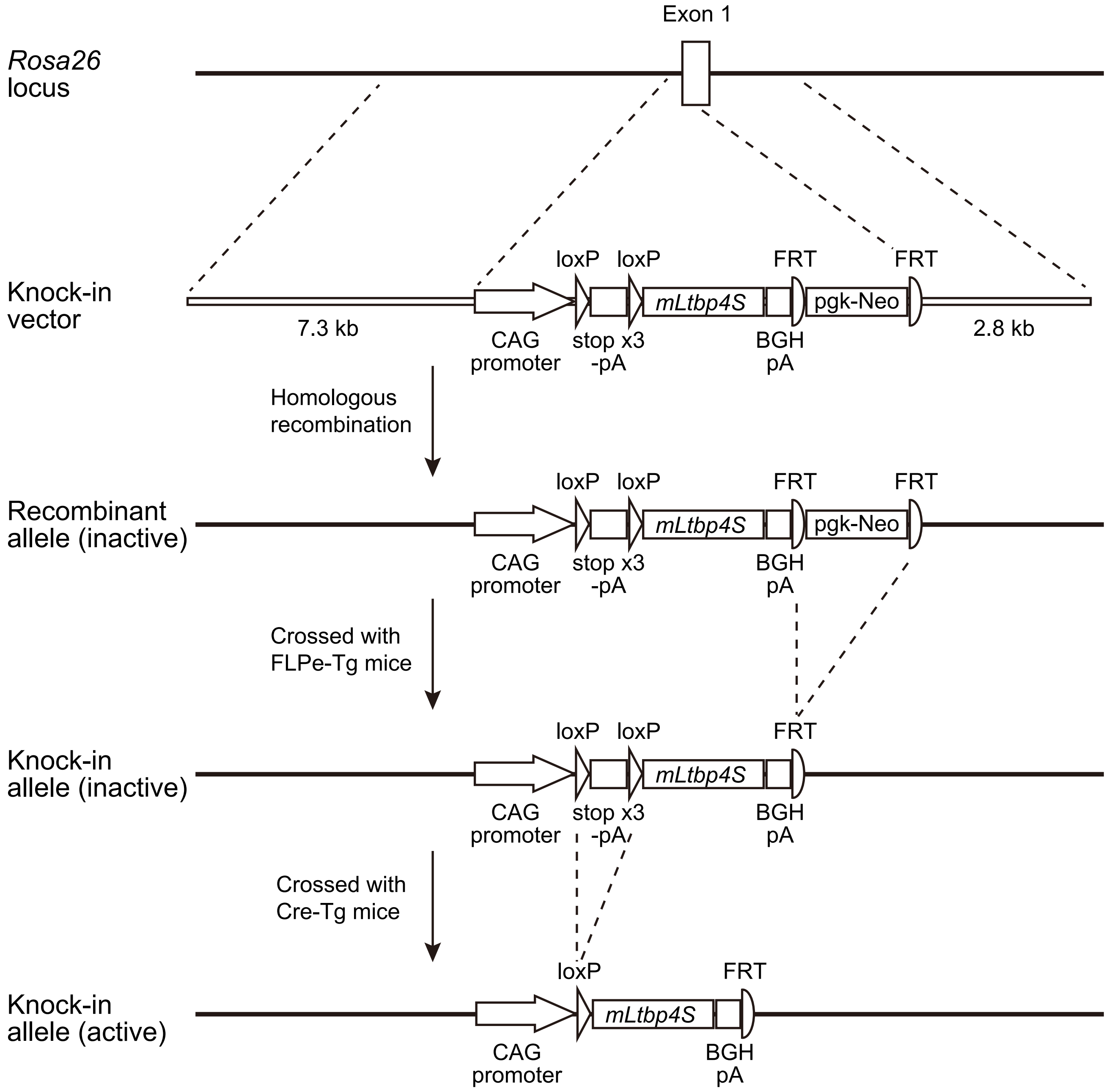

Supplementary Figure S9. Schematic representation of the strategy to generate *Ltbp4S* KI mice. Three stop codons of different frames and a poly-adenylation signal flanked by two loxP sites were inserted between the CAG promoter and *mLtbp4S* cDNA in the knock-in (KI) allele (inactive). The activation of LTBP-4S expression in the whole body by crossing with germline-Cre Tg mice (AyuI-Cre mice) did not cause defects in development and fertility. Therefore, mice with the KI allele (active) were crossed with *Ltbp2* null mice to generate *Ltbp2* null; *Ltbp4S* KI mice.

Supplementary Table S1. Primer sequences for RT-PCR

Ltbp1	forward<br>reverse	5'-GAATGGGCAGTGCAGAAATACCGATGG-3'<br>5'-CGGGCATGTGCAATCATAGGACCCCGC-3'
Ltbp2	forward<br>reverse	5'-AAACCCCTCAGCGACCCGCGGCTGC-3'<br>5'-TGCTTCTGTGAGGACCGGGTGCTCT-3'
Ltbp3	forward<br>reverse	5'-GCAACCCTTTGCCTGGCCTTACCAAG-3'<br>5'-GGGTTAGGCGTGTGGTCAGAGGGTGC-3'
Ltbp4	forward<br>reverse	5'-GCACAAATACTAAAGGCTCCTTCCAC-3'<br>5'-GACACTCGTCAATGTCAAGGCAGGAG-3'
Gapdh	forward<br>reverse	5'-GCTGCCAAGGCTGTGGGCAAGGTCATC-3'<br>5'-TGAGGTCCACCACCCTGTTGCTGTAGC-3'

Supplementary Table S2. Primer sequences for qPCR

Ltbp2	forward<br>reverse	5’-AAACCCCTCAGCGACCCGCGGCTGC-3’<br>5’-TGCTTCTGTGAGGACCGGGTGCTCT-3’
Ltbp4	forward<br>reverse	5’-GCACAAATACTAAAGGCTCCTTCCAC-3’<br>5’-GACACTCGTCAATGTCAAGGCAGGAG-3’
Eln	forward<br>reverse	5’-CTATGGAGGAGCCCTTGGAG-3’<br>5’-CACAGGATTTCCCAAAGCAG-3’
Fn1	forward<br>reverse	5’-GGCTTTGGCAGTGGTCATTTC-3’<br>5’-TTCCATTCCCGAGGCATGT-3’
Fbn1	forward<br>reverse	5’-AATATCTCGGAGCCATTTGC-3’<br>5’-CAGGTCTACGGCAGTTGTCA-3’
Fbn2	forward<br>reverse	5’-TGCAAAATCAATGGCTACACC-3’<br>5’-CTCCAGGCTGATTTGCTCCT-3’
Fbln4	forward<br>reverse	5’-ATGGCTATGAGTGGGATGCAGACAGCCAGC-3’<br>5’-TGGCAATAGCGGTAACGACACTCATCTATG-3’
Fbln5	forward<br>reverse	5′-ATACTCACTGTTACCATTCTGGCT-3′<br>5′-GGTTAACACACATCATGTCTCCTC-3′
Lox	forward<br>reverse	5’-GCCCTCGGTACTCCTGGGAGTGGCACAG-3’<br>5’-AGACAGAAGCTTGCTTTGTGGCCTTCAG-3’
Tgfb1	forward<br>reverse	5’-GCAACAATTCCTGGCGTTACC-3’<br>5’-CGAAAGCCCTGTATTCCGTCT-3’
Tgfb2	forward<br>reverse	5’-AGCTACCTGGGTCCATTCCT-3’<br>5’-GACGCAGAAAAGGCTGAAAC-3’
Tgfb3	forward<br>reverse	5’-CCAGATACTTCGACCGGATGA-3’<br>5’-TGACATCGAAAGACAGCCATTC-3’
Mmp2	forward<br>reverse	5’-CACCTGGTTTCACCCTTTCTG-3’<br>5’-CGAGCGAAGGGCATACAAA-3’
Mmp9	forward<br>reverse	5’-CATGCACTGGGGGCTTAGATCATTC-3’<br>5’-CGAGGGTAGCTATACAGCGGGTAC-3’
Mmp12	forward<br>reverse	5’-TGTACCCCACCTACAGATACCTTA-3’<br>5’-CCATAGAGGGACTGAATGTTACGT-3’
Gapdh	forward<br>reverse	5’-CCATCACCATCTTCCAGGAG-3’<br>5’-CACACCCATCACAAACATGG-3’
